# Cross-sectional study of diabetes kidney disease in the Eastern Cape, South Africa

**DOI:** 10.1097/MD.0000000000023303

**Published:** 2020-12-11

**Authors:** Oladele Vincent Adeniyi, Eyitayo Omolara Owolabi

**Affiliations:** a Department of Family Medicine, Faculty of Health Sciences, Walter Sisulu University/Cecilia Makiwane Hospital, East London; b Centre for Global Surgery, Department of Global Health, Faculty of Medicine and Health Sciences, Stellenbosch University, Cape Town, South Africa.

**Keywords:** diabetes renal disease, dyslipidemia, eastern cape, hypertension, South Africa

## Abstract

Diabetes mellitus (DM) is an independent risk factor for the development of kidney disease. This study assesses the prevalence and determinants of asymptomatic kidney disease in individuals with DM attending health facilities in OR Tambo district, Eastern Cape, South Africa.

In this cross-sectional analysis, medical data of 327 individuals receiving care for DM in primary health care centers in OR Tambo district, Eastern Cape between June and November 2013 were reviewed. Significant kidney disease was defined as estimated glomerular filtration rate less than 60 mL/min/1.73 m^2^ in accordance with the guidelines of the Society of Endocrinology, Metabolism and Diabetes of South Africa (2017).

One-quarter of the 327 participants (n = 80) had significant kidney disease. Female sex [odds ratio (OR) = 5.2; 95% confidence interval (95% CI) 1.2–23.5], never used alcohol (OR = 13.4; 95% CI 2.5–72.1), hypertension (OR = 16.2; 95% CI 2.0–130.0), triglyceride (TG)/high-density lipoprotein (HDL) ratio (OR = 1.2; 95% CI 1.0–1.5), current smoker (OR = 1127.9; 95% CI 162.9–7808.9), former smoker (OR = 13.3; 95% CI 4.1–41.4), and longer duration of diabetes (OR = 4.6; 95% CI 1.6–13.0) were the independent determinants of significant kidney disease among the participants. A significant dose--effect relationship exists between renal disease and smoking status (*P* < .0001), duration of DM (*P* < .001), glycemic status (*P* = .025), and body mass index (*P* = .003).

There is a high rate of undiagnosed kidney disease in this setting, which was independently associated with female sex and presence of other cardiovascular risk factors. Strategic interventions targeting screening and monitoring of renal functions in individuals with DM are urgently needed in this region.

## 1 Introduction

Chronic kidney disease (CKD) is one of the most common complications associated with diabetes.^[1]^ It is found in one-third of individuals with diabetes mellitus (DM), and a leading cause of end-stage kidney disease (ESKD).^[1–3]^ In addition, CKD contributes significantly to increased risk for various cardiovascular events and all-cause mortality, making it a disease of significant public health concern.^[4,5]^ Worryingly, CKD is mostly asymptomatic, thus, leading to delayed diagnosis and treatment, and consequently, its progression to ESKD^[6–8]^ and cardiovascular mortality.^[4,9]^ As such, early identification through regular screening, prompt treatment, as well as adherence to treatment are crucial to slow down the progression and mitigate the associated burden.^[10,11]^ Yet, several studies have highlighted a low level of diagnosis, awareness and treatment of CKD among its sufferers.^[12–14]^

In recognition of the importance of prompt diagnosis and management of CKD, there is a constant advocacy for regular screening of patients, especially those living with diabetes or hypertension, which poses a higher risk.^[15]^ The Society for Endocrinology, Metabolism and Diabetes of South Africa recommended screening for CKD in individuals with DM at diagnosis and subsequently, annually.^[16]^ Such screenings are essential in order to promptly detect and initiate necessary management plans to delay progression and further guide clinicians to adjust antidiabetic drugs.^[4,9,14,16,17]^ CKD is associated with poor glycemic control and overall treatment outcomes.^[4,9,17]^

South Africa is confronted with a quadruple burden of disease, which places significant strain on the health care system and an increase in the burden of CKD and its associated cardiovascular risks will further increase the strain on the healthcare system.^[18,19]^ Also, the presence of such complications increases the health care costs and negatively impact the individual's health-related quality of life.^[20–22]^ Few studies have identified lack of resources for diagnostic screening as a major barrier to early detection of CKD.^[11,23,24]^ Even in the presence of resources in some settings, clinicians often do not follow guideline recommendations on screening for CKD in at-risk individuals.^[25,26]^ There also exist patients’ factors such as delayed presentation and lack of compliance with recommended treatment, which impacts on the progression of CKD.^[11,27]^ In addition, demographic factors such as age, level of education, and gender have also been implicated to influence CKD diagnosis and management.^[10]^ The situation in South Africa, like many other developing countries, is further compounded by a lack of epidemiological data,^[11,23,24]^ which sometimes lead to underestimation of the burden of CKD. Such epidemiological data are crucial in designing appropriate interventions.

We hypothesized that significant proportions of individuals in care for DM may be having undiagnosed significant kidney disease, which might impact on treatment and glycemic control. This study therefore sought to determine the prevalence and correlates of asymptomatic kidney disease in individuals with DM in OR Tambo district, Eastern Cape, South Africa. The Eastern Cape is a resource-constrained province with a high burden of chronic diseases and suboptimal treatment targets.^[28,29]^ The findings of this study will guide clinicians in the care for people with DM and crafting of context-specific interventions in the province.

## 2 Methods

### 2.1 Study design and setting

In this study, we conducted cross-sectional analysis on data of 360 adults (age ≥30 years) attending chronic care for DM in OR Tambo district health facilities. The detailed methods of the primary study had been published previously.^[29]^ Briefly, individuals attending community health centers in OR Tambo district and Mthatha Regional Hospital for DM between June and November 2013 were recruited. Mthatha Regional Hospital provides both level 1 and 2 services and provide support to the community health centers in the district. Of the total participants (N = 360), complete medical data were not available for 33 participants, hence, were excluded in this analysis.

### 2.2 Ethical approval

The protocol for the study was approved by the Ethics Committee of Walter Sisulu University (Reference: 031/2013), and permission was granted by the Eastern Cape Department of Health and clinical governance of the hospital. Written informed consent was obtained from each participant, indicating his/her voluntary participation and permission to share the findings of the study. The research process respected the right to privacy and confidentiality of medical information in accordance with the Helsinki Declaration.

### 2.3 Data collection

Data sources include personal interview, review of medical records, anthropometry measurements, and blood assays of participants. Medical records of the participants on treatment for DM for at least a year were reviewed. Additional data were obtained by direct interview of the participants on their smoking status, alcohol use, and level of physical activity. History of hypertension and duration of DM (from time of diagnosis) were extracted from the medical records. Lifestyle behaviors were further categorized based on frequency of use of the cigarette and alcohol. Smoking status was categorized as never smoked, former smoker (if already quit smoking for at least a month), and current smoker. Alcohol use was categorized as never drank and current drinker (if still consuming alcohol). Regular engagement in activities associated with increase in heart rate and respiratory rates was defined as being active.

Using a mounted stadiometer, we measured the height of each participant to the nearest 0.1 m without shoes in a standing position with feet together. We also weighed the participants in light clothes with a digital scale (Tanita-HD 309; Creative Health Products, MI) to the nearest 0.1 kg. We estimated the body mass index (BMI) as the weight in kg divided by height in square meters (kg/m^2^). The BMI was categorized as obese if the BMI ≥30.0 kg/m^2^ and further classified as mild obesity (BMI = 30.0–34.9 kg/m^2^), moderate obesity (BMI = 35.0–39.9 kg/m^2^), and morbid obesity (BMI ≥ 40 kg/m^2^) in accordance with the World Health Organization.^[30]^

After overnight fast for 8 hours without food, the investigator drew 5 mL of venous blood sample for creatinine, total cholesterol (TC), triglycerides (Trig), high-density lipoprotein cholesterol (HDL-C), and low-density lipoprotein (LDL-C). All laboratory assays were performed by the National Health Laboratory Services in accordance with the standard protocols.

Glomerular filtration rate (main outcome variable) was estimated by using the CKD epidemiology equation. Significant kidney disease was defined as estimated glomerular filtration rate (eGFR) less than 60 mL/min/1.73 m^2^ in accordance with the guidelines of the Society of Endocrinology, Metabolism and Diabetes of South Africa.^[16]^

### 2.4 Statistical analysis

Data were entered into an excel spreadsheet and cleaned. Analysis was completed using the Statistical Package for Social Science (SPSS) version 24.0 for Windows (SPSS, Chicago, IL). Data on continuous variables were expressed as mean values ± standard deviations (SD). Counts (frequency) and proportions (percentages) were reported for categorical variables. Using the Maentel--Haenszel test, the univariate odds ratios (ORs) were used to examine the variables that have significant associations with kidney disease. The multivariate ORs and their 95% confidence intervals (95% CIs) were estimated by using logistic regression analysis to identify the independent determinants of kidney disease after adjusting for confounding factors. In addition, the direction of association of the determinants with the worsening of the creatinine level was examined with the plot logistic regression analysis. A *P*-value < .05 was considered statistically significant.

## 3 Results

The majority of the participants were at least 50 years old (n = 248; 75.8%). Most of the participants were females (70.3%), reside in rural areas (88.7%), never drank alcohol (64.5%), living with DM for less than 10 years (24.5%), have concomitant hypertension (81.1%), have sedentary lifestyle (67.0%), and are obese (60.2%). There was low HDL-C (atherogenic dyslipidemia) in 45.6% and non-LDL related dyslipidemia in 17.7% of the participants (Table 1).

**Table 1:** Univariate significant determinants of kidney disease.

	All	Presence of kidney disease		
Variables	n (%)	n (%)	OR (95% CI)	*P*
Gender				
Females	230 (70.3)	67 (29.1)	2.7 (1.4–5.1)	.003
Males	97 (29.7)	13 (13.4)	1	
Monthly income				
≥ R1000	237 (72.5)	66 (27.8)	2.1 (1.1–4.0)	.021
< R1000	90 (27.5)	14 (15.6)	1	
Alcohol use				
Never drank	211 (64.5)	64 (30.3)	2.7 (1.5–5.0)	<.001
Current drinker	116 (35.5)	16 (13.8)	1	
DM duration, yr				
≥ 10	80 (24.5)	31 (38.8)	2.6 (1.5–4.4)	<.001
< 10	247 (75.5)	49 (19.8)	1	
Hypertension				
Yes	265 (81.1)	74 (27.9)	3.6 (1.5–8.8)	.003
No	62 (18.9)	6 (9.7)	1	
Physical activity				
Inactive	219 (67.0)	65 (29.7)	2.6 (1.4–4.9)	.002
Active	108 (33.0)	15 (13.9)	1	
Low HDL-C				
Yes	149 (45.6)	72 (48.3)	20.0 (9.1–43.2)	<.0001
No	178 (54.4)	8 (4.5)	1	
Non-LDL-related dyslipidemia				
Yes	58 (17.7)	27 (46.6)	3.6 (2.0–6.5)	<.0001
No	269 (82.3)	53 (19.7)	1	
Obesity				
Yes	197 (60.2)	63 (32.0)	3.1 (1.7–5.7)	<.0001
No	130 (39.8)	17 (13.1)	1	

CI = confidence interval, DM = diabetes mellitus, HDL-C = high-density lipoprotein cholesterol, LDL-C = low-density lipoprotein cholesterol, OR = odds ratio.

### 3.1 Prevalence of asymptomatic kidney disease

Of the total participants (N = 327), 80 participants had underlying asymptomatic kidney disease (prevalence of 24.5%), which differ significantly by sociodemographic and clinical parameters (Table 1).

### 3.2 Determinants of asymptomatic kidney disease by univariate analysis

Gender, monthly income, alcohol use, duration of DM, presence of hypertension, obesity, and physical activity were all significantly associated with the presence of asymptomatic kidney disease. There was significant association between atherogenic dyslipidemia (low HDL-C and non-LDL related dyslipidemia) and presence of asymptomatic kidney disease (Table 1). The likelihood of developing kidney disease was 3-fold for female, 2-fold for individuals earning R1000 or more, 3-fold for individuals who never drank alcohol, 3-fold for those who had DM for longer period (≥10 years), 20-fold for individuals with sedentary lifestyle and hypertension (4-fold). Similarly, there is a higher likelihood of developing kidney disease among patients with low HDL-C (20-fold), non-LDL related dyslipidemia (4-fold), and obese (3-fold).

### 3.3 Determinants of asymptomatic kidney disease by multivariate logistic regression (model) analysis

In the multivariate logistic model analysis, female sex, nonalcohol use, smoking, hypertension, TG/HDL ratio, and longer duration of diabetes were independently and significantly associated with asymptomatic kidney disease among the participants. Female patients were 5 times more likely to have asymptomatic kidney disease than male patients with DM. Patients who never had history of alcohol use were 13 times more likely to have kidney disease than those with history of alcohol use. Similarly, patients with concomitant hypertension are 16 times more likely and those with DM for at least 10 years are about 5 times more likely to have asymptomatic kidney disease. A significant dose--effect response relationship was observed between kidney disease and the smoking history of the patients. The odds of developing kidney disease are significantly very high among current smokers than former smokers in comparison with nonsmokers (Table 2).

**Table 2:** Independent determinants of asymptomatic kidney disease by logistic regression analysis.

Variables	B	SE	Wald	OR (95% CI)	*P*
Gender					
Female	1.657	0.764	4.697	5.2 (1.2–23.5)	.030
Male				1	
Alcohol use					
Never drank	2.592	0.860	9.077	13.4 (2.5–72.1)	.003
Current drinker				1	
Hypertension					
Yes	2.785	1.063	6.866	16.2 (2.0–130.0)	.009
No				1	
TG/HDL ratio					
≥ 1.5	0.219	0.087	6.319	1.2 (1.0–1.5)	.012
< 1.5				1	
Smoking status					
Current smoker	7.028	0.987	50.684	1127.9 (162.9–7808.9)	<.0001
Former smoker	2.562	0.592	18.734	13.3 (4.1–41.4)	<.0001
Never smoked				1	
Duration of DM, yr					
≥ 10	1.530	0.529	8.373	4.6 (1.6–13.0)	.004
< 10				1	

CI = confidence interval, DM = diabetes mellitus, HDL = high-density lipoprotein, OR = odds ratio, SE = standard error, TG = triglyceride.

**Figure 1:**
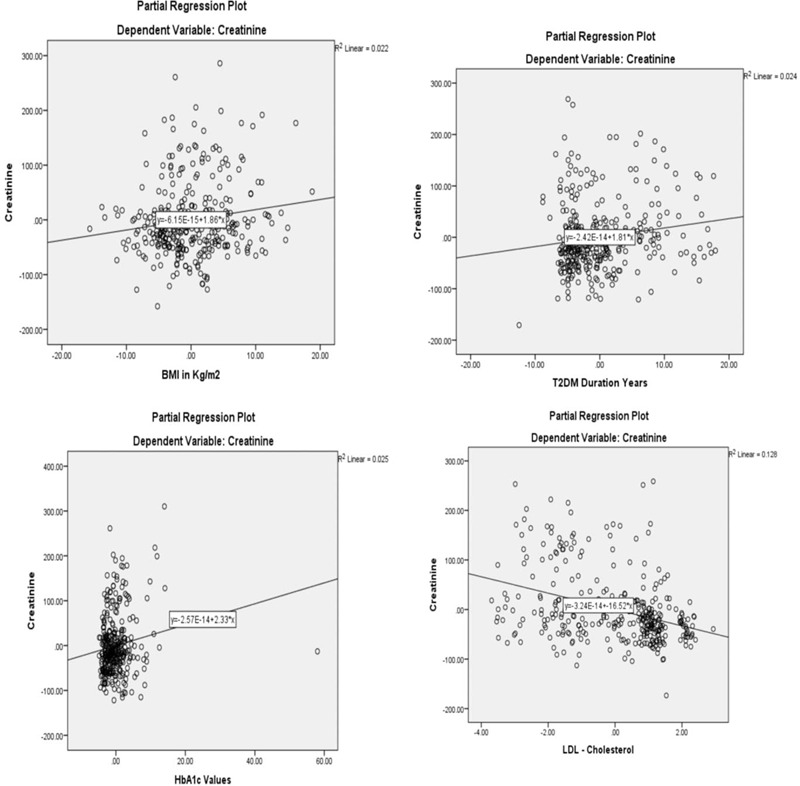
Linear relationship between clinical parameters and presence of kidney disease.

There was a significant positive association (*P* for trend < .0001) across the groups of never smoked, former smokers, and current smokers and kidney disease among the participants. Similar trends were observed between the duration of DM (*P* < .001), increasing HbA1C (*P* = .025), increasing BMI (*P* = .003), and increasing creatinine levels in the participants. However, a negative association was observed between increasing levels of LDL-C and creatinine level in the patients (*P* = .120) (Fig. 1).

### 3.4 Current medications in individuals with underlying kidney disease

All the participants were at least on either insulin or oral hypoglycaemic drugs or both. Significantly higher proportion of the participants (96.9%, n = 317) were treated with metformin alone or in combination with insulin therapy, and/or sulphonylureas. Among those with asymptomatic kidney disease, 98.7% (n = 79) were on metformin (Table 3).

**Table 3:** Current medications in patients with asymptomatic kidney disease (N = 80).

Variables	Frequency (n)	Percentage (%)
Insulin alone	22	27.5
Metformin	79	98.8
Glibenclamide	21	26.3
Gliclazide	25	31.3
Metformin + Insulin	23	28.8
Gliclazide + Insulin	3	3.8

## 4 Discussion

Given the asymptomatic nature of diabetes kidney disease in the early stages, clinicians need to monitor either the presence of albuminuria or eGFR in individuals with DM.^[16]^ This study sought to determine the prevalence and correlates of asymptomatic kidney disease in individuals with DM in OR Tambo district, Eastern Cape, South Africa. The findings of this study might shed light on the practice gaps in the care for patients with DM and inform the crafting of local protocols for clinicians in the district.

This study found that 1 in every 4 individuals with DM (24.5%) in this setting had asymptomatic kidney disease. This finding calls for concern, given the quadruple burden of disease existing in South Africa competing for the limited resources required to manage ESKD.^[11]^ The prevalence reported in this study is similar to previous report by Janmohammed^[11]^ among patients with DM. The finding in this study further elucidate the importance of routine screening for kidney disease in individual with DM at diagnosis and annually.^[16]^

The study finds a linear relationship between glycemic control and creatinine level; worsening glycosylated hemoglobin and deterioration of kidney function in the cohort. The finding of this study also reflects the poor glycemic control in individuals with DM in the region.^[29]^ Prompt detection and treatment of kidney disease are essential for optimization of glycemic outcomes. Also, it is critical for the reduction in the risk for diabetic complications or the worsening thereof.^[17]^ As summarized in Table 3, nearly all the patients with kidney disease (98.8%) were treated with metformin. Given that metformin is excreted in the kidney, there is evidence supporting the withdrawal of this drug in patients with kidney compromise especially at eGFR <30 mL/min/1.73 m^2^.^[16,17]^ Lactic acidosis is a known complication following accumulation of metformin in patients with kidney failure.^[31]^ Therefore, clinicians need to monitor the patients with DM in order to switch to safer drug options.

Furthermore, females have higher odds for developing kidney disease in comparison with their male counterparts. The impact of gender on CKD remains controversial. Although some studies documented higher risk of developing CKD among women,^[10,32,33]^ others reported higher odds among men.^[34–37]^ Several plausible biological explanations have been advanced on the protective effect of the female gender on CKD such as the difference in the anatomical structure and the hemodynamic response of the kidney to stress and the effect of the female sex hormones on the development of CKD.^[38,39]^ Cherney et al^[40]^ posited that women exhibit reduced kidney blood flow and an increase in kidney vascular resistance and filtration fraction when blood sugar is raised, but men do not exhibit such hemodynamic changes, which explain the probable lack of renal protection among diabetic women. Another probable explanation is the gender difference in the lifestyle behaviors and the resultant cardiometabolic effects.^[41,42]^ Some of the cardiovascular risk factors (obesity and physical inactivity) reported in this study as independent determinants of CKD are often found at higher rate among women than men in this population.^[43,44]^

Also, the presence of cardiovascular risk factors such as hypertension, dyslipidemia, obesity, and smoking were associated with a higher odds for developing CKD. This is not surprising, given the roles these risk factors play in the progression and treatment outcomes of diabetes management and vice versa.^[45]^ Overweight and obesity are independent risk factors for CVD mortality,^[46]^ and contribute to the development of metabolic risk factors such as diabetes, dyslipidemia, and hypertension.^[47,48]^ Also, high blood pressure can lead to damage of blood vessels, which reduce blood flow to several organs, including the kidney. In addition, high blood pressure could also affect the glomerular filtration, which leads to accumulation of body wastes and fluid in the body, which further raises blood pressure and further damage the kidney.^[49]^ Likewise, the presence of hypertension in individuals with DM worsen glycemic control and thus, increased risk for kidney function decline.^[50]^

Similar to the influence of high blood pressure on the incidence of CKD in individuals with DM, dyslipidemia (high TC, high LDL-C, and low HDL-C) also contribute to the incidence and progression of CKD and vice versa.^[51]^ Dyslipidemia is associated with a decline in kidney function and it is a common complication of CKD. For instance, there is an associated increase in TG among patients with CKD as a result of the reduced catabolism and the increase in the production of hepatic triglyceride-rich lipoproteins.^[52,53]^ There is also an associated reduction in the sizes and density of LDL and reduced production of HDL-C in the presence of compromised renal function. These all contribute to the atherosclerosis, which puts the individual at a higher risk of cardiovascular events.^[53,54]^

Advancement in age is also associated with a higher incidence of insulin resistance and impaired insulin secretion as a result of impairment in the functioning of the islet cell,^[55,56]^ which negatively impacts glycemic control and consequently, CKD development. This is a probable explanation for the observed relationship between duration of illness and CKD in this study. The older individuals will likely constitute those with longer duration of illness and thus, a higher risk for cardiovascular events and poor glycemic control, while nonadherence with treatment could underlie poor treatment outcome among those newly diagnosed of diabetes.

The study found a significant association between smoking status and worsening kidney function. Cigarette smoking independently contributes to the development of CKD.^[57]^ It causes decline in kidney function and is associated with elevated CRP, an inflammatory marker.^[57,58]^ Smoking cessation is of great clinical importance in individuals with DM due to the synergistic interaction between smoking and other cardiovascular risk factors, and causes deterioration of kidney functions through its effects on albumin excretion, thus, increasing the risk of micro-albuminuria.^[59]^

Alcohol use was associated with lesser odds of developing CKD. There are contradictory reports on the impact of alcohol use on cardiovascular health, blood sugar, and CKD. Although some studies have documented an inverse association between alcohol use and CKD,^[60,61]^ others have reported no effect on CKD development,^[62]^ and some studies reported positive impact of moderate alcohol use on CVD and glycemic status.^[63,64]^ It should be noted that the amount of alcohol use (whether moderate or heavy alcohol use) by the participants could have provided more information on the impact of alcohol on the kidney functions. However, there is no safe limit on the use of alcohol.^[65–67]^ The underlying mechanism for the impact of moderate alcohol on CKD was the associated increase in insulin sensitivity^[68]^ and increased serum HDL-cholesterol.^[69,70]^ A J-shaped relationship between alcohol use; a beneficial effect at low to moderate alcohol use in comparison to deleterious effect at heavy alcohol use have been reported. This reno-protective effect was reported in individuals with or without underlying kidney disease.^[71,72]^ At low to moderate amount, alcohol consumption is associated with the formation of less amount of hyaline in the renal arterioles.^[73]^ In addition, polyphenols and quercetin (found in wine) have been shown to reduce cyclosporin associated nephrotoxicity in animals.^[74]^ Also, alcohol tends to induce antioxidant enzymatic activities, which thus protect against oxidative stress-dependent kidney damage.^[75]^ More studies are needed to elucidate on the complex relationship between alcohol (at different levels of use) and CKD.

### 4.1 Limitations of the study

The study limitations cannot be ignored, given that only a single eGFR was analyzed in this study; as such, the extent of CKD could not be ascertained. In addition, the urine albumin creatinine ratio would have complemented the eGFR if available for analysis in this study. In addition, family history of CKD was not obtained in this study, which could have elucidated on the relationship between genetics and CKD in the cohort. However, the findings gave credence to the importance of regular monitoring of kidney functions in individuals with DM and/or hypertension, especially in resource-constrained settings. Also, the study highlights the need for clinicians to adhere to guideline recommendations on screening for diabetes kidney disease.

## 5 Conclusion

There is a high rate of undiagnosed kidney disease in this setting, which was independently associated with female sex, hypertension, dyslipidemia, cigarette smoking, longer duration of DM, and poor glycemic control. Clinicians must address all these cardiovascular conditions in addition to optimizing glycemic control in individuals with DM. This study recommends periodic audit of clinical care of patients with DM in the district in order to ensure compliance with evidence-based guideline recommended by the Society of Endocrinology, Metabolism and Diabetes of South Africa.

## Acknowledgments

The authors are grateful to the management and staff of Mthatha Regional Hospital for the successful completion of this project.

## Author contributions

**Conceptualization:** Oladele Vincent Adeniyi.

**Data curation:** Oladele Vincent Adeniyi, Eyitayo O. Owolabi.

**Formal analysis:** Eyitayo O. Owolabi.

**Funding acquisition:** Oladele Vincent Adeniyi.

**Investigation:** Oladele Vincent Adeniyi.

**Methodology:** Oladele Vincent Adeniyi.

**Project administration:** Oladele Vincent Adeniyi.

**Resources:** Oladele Vincent Adeniyi.

**Software:** Oladele Vincent Adeniyi.

**Supervision:** Oladele Vincent Adeniyi.

**Validation:** Oladele Vincent Adeniyi.

**Writing – original draft:** Oladele Vincent Adeniyi, Eyitayo O. Owolabi.

**Writing – review & editing:** Oladele Vincent Adeniyi, Eyitayo O. Owolabi.
